# Supplementary material for: “I bought it, but I still don’t know what it is”: international students dealing with a new food culture in Norway − A qualitative study
Source: Int J Qual Stud Health Well-being. 2025 Dec 4;20(1):2595849. doi: 10.1080/17482631.2025.2595849 (PMC12683748; doi:10.1080/17482631.2025.2595849)
Supplement: Supplementary Material — Clean copy - Manuscript_with_author_details_clean_version_141125.docx [file ZQHW_A_2595849_SM4280.docx]

**“I bought it, but I still don’t know what it is”: International students dealing with a new food culture in Norway ­− A qualitative study**

Anine C. Medin ^1,^*, Maaike E. M. Polspoel ^1,2^, Kristine Vejrup ^1,3^, Sofia T. Strömmer ^4,5^, Mary E. Barker ^1,4,5^, and Nina C. Øverby ^1^

^1^ Department of Nutrition and Public Health, Faculty of Health and Sport Sciences, University of Agder, P.O. Box 422, 4604 Kristiansand, Norway.

^2^ Faculty of Medicine and Health Sciences, Ghent University, Gent, Belgium.

^3^ Norwegian Armed Forces Medical Services, Sessvollmoen, Norway.

^4^ MRC Lifecourse Epidemiology Unit, University of Southampton, Southampton General Hospital, Southampton, UK.

^5^ NIHR Southampton Biomedical Research Centre, University of Southampton and University Hospital Southampton NHS Foundation Trust, Southampton, UK.

***** Corresponding author: [anine.medin@uia.no](mailto:anine.medin@uia.no)

# Abstract

**Background:** Worldwide, more than six million students study abroad each year, many of whom experience culture shock. Engaging with a new and different culture often changes food choices and eating habits, and the process of dietary acculturation is known to be difficult for young people moving abroad, away from family and home culture. Despite this, little is known about the dietary acculturation of international students in the Nordic context and how it affects their health and well-being. This qualitative study aimed to gain insight into the main challenges international students face concerning food choices and eating habits after encountering Norwegian food culture.

**Methods:** International students (n=10) attending a university in Southern Norway, were interviewed using semi-structured face-to-face interviews. Inductive codebook thematic analysis within an applied orientation was used to analyse the data.

**Results:** Five main themes were identified: food cost, missing the taste from home, food literacy, language barriers, and the social aspects of eating. Food cost was identified as the most significant challenge, possibly influencing food choices and eating habits, and it seemed to be particularly critical for non-European students.

**Conclusions:** This study indicated that international students in Norway encounter several food-related challenges, such as those faced by their peers in other countries. These challenges may have a negative impact not only on the quality of their diet but also on their social and emotional well-being. These insights provide valuable perspectives on the social, emotional, and practical issues of adapting to a new culture, potentially informing measures of support for international students in the Nordic context.

**Keywords:** international students; eating habits; food culture; well-being; dietary acculturation; cultural adaptation.

# 1. Introduction

Each year, over six million university students from around the world study abroad (Migration Data Portal, 2024). In Norway alone, international students comprised 8% of the student population in 2023 (Statistics Norway, 2023; Norwegian Directorate for Higher Education and Skills, 2024). These students are not only an important revenue source for universities (Wiers-Jenssen, 2019), but also contribute to the cultural diversity of student communities. Therefore, keeping these students happy, well, and healthy is in the interest of universities, both academically and economically.

While studying abroad can empower students by increasing their intercultural understanding and leading to significant academic and personal growth (Roy et al., 2019), it can also present challenges. One such challenge is culture shock, which is the anxiety caused by losing familiar ways of communication and social interaction (Oberg, 1960). Other observed issues among international students include negative effects on their phyco-social well-being such as loneliness and homesickness, leading to social isolation and stress (Arthur, 2017; Poyrazli and Lopez 2007; Heng 2016).

Acculturation, the process experienced when individuals encounter a new culture, involves significant personal transition and cultural adaptation. Redfield’s classic definition describes acculturation as a phenomenon resulting from continuous contact between groups of individuals from different cultures, leading to changes in the cultural patterns of either or both groups (Redfield, 1936, p. 149).

Food and eating are both crucial to our existence and are integral to our cultural identity; therefore, they play a central role in acculturation. The process by which individuals adopt the food culture and eating habits in a new country is referred to as dietary acculturation. According to Satia’s framework, dietary acculturation is a complex, nonlinear process influenced by multiple factors, including individual characteristics, socio-economic status, ethnocultural norms, and exposure to the host culture. These influences include factors such as age, country of origin (including urban vs rural background), education, knowledge, attitudes and beliefs, taste preferences, food availability, affordability, and social support (Satia, 2010).

Dietary acculturation and diet quality have been researched extensively in the context of immigration to Western countries, typically showing a shift toward poorer dietary patterns among most ethnic groups following migration (Gilbert & Khokhar, 2008; Sanou et al., 2014). Notably, the negative impacts differ between immigrants in the United States (US) and Canada compared to Europe, where they seem less severe (Popovic-Lipovac & Strasser, 2015).

Research on dietary acculturation among international students has largely been conducted in the US, Canada, and the United Kingdom (UK), echoing findings from immigrant studies. A recent US study involving international students from 41 countries reports a decline in their diet quality with increased consumption of junk food and reduced intake of whole foods, including fruits and vegetables (Dean et al., 2024).

Several studies have observed that dietary acculturation among international students not only is associated with reduced diet quality but is also associated with negative effects on their health and well-being. For instance, a study including international students in the US demonstrated that they had challenges related to their diets due to cultural adaptation, which was associated with physical health issues such as weight gain and high blood pressure as well as negative mental health outcomes (Alakaam et al., 2015). Interviews with international students in the UK have shown that these students feel a loss of social bonding, typically associated with mealtimes, and found food from their home countries to be emotionally comforting (Brown et al., 2010). Similarly, findings from a study from Canada, using focus group discussions and photo-voice, indicate that international students miss both foods from their home countries and cultures and the social aspects of the eating (Amos & Lordly, 2014).

These studies illustrate that dietary acculturation affects the social, psychological, and physical health and well-being of international students. A recent study further supports this perspective, arguing that food serves as a critical yet often overlooked method of acculturation for international students due to its substantial influence on personal, psychological, and social domains (He et al., 2024). It demonstrates how familiar foods provide comfort and relief, while unfamiliar foods intensify feelings of isolation, homesickness, and stress, affecting overall well-being. Additionally, according to Ha et al., food can both facilitate and hinder acculturation, serving as a connecting medium but also a way to discriminate and stereotype, thereby negatively affecting international students’ well-being.

There is a notable gap in the literature regarding dietary acculturation among international students in Europe, outside the UK, both as an independent topic and in relation to well-being. One of the few identified exceptions is a study protocol for a food education intervention in Italy targeting international students (Neri et al., 2025). In the Nordic region, the literature is similarly scarce. Only one small qualitative study has examined food choices and food insecurity among international students in Norway. It reported that these students struggled to maintain a varied diet in line with their preferences, and that their social eating was negatively impacted (Bauch et al., 2023). Given this research gap, particularly within the Nordic context, this study aimed to explore the experiences of international students in Norway, focusing on the main challenges they face in relation to food and eating habits as they encounter the local food culture, with potential implications for their health and well-being. This emphasis reflects prior research showing that dietary difficulties are common among international students in other countries.

# 2. Materials and Methods

## 2.1. Study design and analytic orientation

We conducted an inductive, codebook thematic analysis in an applied tradition. This approach is characterized by data-driven coding, and the iterative development of a shared codebook to guide a team-based analysis. Our procedure aligns with the codebook thematic analysis family described by Braun and Clarke (Braun & Clarke, 2022), distinct from reflexive thematic analysis, and reflects practices commonly used in applied qualitative research (Guest, MacQueen & Namey, 2012). We followed Braun and Clarke’s original six-phase process (Braun & Clarke, 2006) as a broad guide while adapting steps pragmatically for a multi-coder team. The COnsolidated Criteria for REporting Qualitative Research (COREQ) checklist was used as a reporting aid to enhance transparency where applicable.

## 2.1. Study setting and context

This study was conducted at the University of Agder (UiA) in Southern Norway during early spring 2020, before the COVID-19 outbreak in Norway and the subsequent lockdown of university campuses. At any given time, a few hundred international students study at UiA. In 2023, international students comprised 3.4% of 13,861 students (University of Agder, 2023). University housing, often equipped with shared kitchens, is available, along with campus canteens and cafés, where students can purchase meals, including warm lunch or dinner. Norwegian food culture traditionally revolves around two or three cold, bread-based meals (breakfast, lunch, and supper) and one warm, fish- or meat-based main meal (dinner) (Bugge & Almås, 2006). The academic year and autumn semester start in August, while the spring semester begins in January. International students at UiA can start their studies in either autumn or spring, with most staying for one or two semesters.

## 2.2. Recruitment

Participants were eligible if they were international students aged 18 years or older and were studying for a bachelor's or master's degree at UiA.

Participants were initially recruited in person using convenience sampling at social events, which were open to all international students at UiA and organized by the local student network (European Student Network). Recruitment was conducted by M.E.M.P., who was a European international student at the time (age 31 years). After the first recruitment of six participants, five of whom were European students, we transitioned to a purposive sampling approach to recruit additional non-European students at the social events, to ensure a more varied representation. In total, 19 international students were invited to participate, and ten consented to be interviewed. Half of the participants were European students. At the time of the data collection in March, all participants in the current study were relatively new to Norway. They had either arrived within the last three months at the semester’s start in January or were in their second semester, allowing us to capture experiences from students with approximately 2-7 months of exposure to Norwegian culture.

Table 1 describes the participants’ gender, age, and continent of origin.

**Table 1.** Characteristics of the participants

| **No** | **Gender** ^1^ | **Age, years** | **Continent of origin** |
| --- | --- | --- | --- |
| 1 | Woman | 21 | Europe |
| 2 | Man | 24 | Europe |
| 3 | Man | 29 | Europe |
| 4 | Woman | 20 | Europe |
| 5 | Man | 19 | Europe |
| 6 | Man | 21 | Asia |
| 7 | Woman | 26 | Asia |
| 8 | Man | 21 | South America |
| 9 | Woman | 24 | Asia |
| 10 | Man | 24 | Asia |

^1^ Participants reported their gender as either man or woman.

## 2.3. Data collection

Individual in-depth interviews were conducted in February and March 2020. Information about the study was provided before the interviews. Signed written informed consent was obtained from all participants, and no incentives were offered to participate in the study. The interviews were conducted and transcribed by the same researcher (M.E.M.P.) with the support of a team with expertise in interview techniques and qualitative methods. A semi-structured interview guide was used, covering the following topics: food habits in participants' home countries and in Norway, changes in their eating habits since arriving in Norway, and opinions about food purchasing and the availability of different foods in Norway. All interviews were conducted in English in a common lounge area at the university, sufficiently spacious to ensure privacy. The interviewer had no prior relationship with any of the participants prior to the interview. The interviews lasted 20-30 minutes and were audio-recorded using a handheld audio recorder (Zoom H1n). Field notes were not used in this study. Data saturation was discussed continuously during data collection between M.E.M.P. and A.C.M. (project leader), and recruitment was discontinued at n=10 when no new topics were discovered from the interviews.

## 2.4. Data analysis

Data were anonymized prior to analysis. An inductive codebook thematic analysis within an applied orientation (Braun & Clarke, 2022; Guest, MacQueen & Namey, 2012) was used. While the interviews covered a range of experiences, themes primarily reflected challenges; positive aspects were noted but not developed into themes. Three researchers (K.V., A.C.M., M.E.M.P.) from the research team conducted the analyses. First, the researchers familiarized themselves with the data by reading and re-reading all the interviews. Subsequently, three interviews were coded separately by all three researchers to create an initial set of codes. Through discussions we constructed an initial codebook which guided, but did not determine, the subsequent coding for the rest of the interviews. The codebook was iteratively refined as coding progressed, with differences in interpretation resolved through negotiated meaning. After coding all interviews, codes were organized into candidate themes and refined through further discussions and by revisiting the codes. NVivo 12 PRO was used to assist with data handling and as a tool for analysis.

## 2.5. Ethics and approvals

The study was approved by the Norwegian Centre for Research Data (reference number: 923 564) and the Ethical Board of the Faculty of Health and Sport Sciences at the University of Agder, Norway (reference number: RITM0064045).

# 3. Results

## 3.1. Overall themes

Five main themes were identified, all describing food-related challenges that resulted in changes in eating habits for international students since their arrival in Norway. These changes were sometimes for the worse and occasionally for the better. Themes and subthemes are described below, along with illustrative quotes.

## 3.2. Theme one: Food cost

The theme of “Food cost” was of considerable importance to the participants, as it was mentioned by every international student, regardless of their country of origin. Challenges related to food costs seemed greater for non-European students than for European students.

### 3.2.1. Food is expensive

All the students talked about the high prices of food and beverages, including alcohol. There was a distinct difference between European and non-European students in this sample in terms of how much they were affected. For non-Europeans, expensive food led them to ration what they could buy and resort to foods that they would not normally eat in their home countries.

*“The food here in the shops is really expensive! So, I look for the cheaper options… Like rice and pasta. Pasta is something I usually don’t eat…”* (Woman, 26 years, from Asia).

One of the consequences of the high cost of food was that it often led students to eat less varied diets; for some, the high prices of food not only restricted what they bought, but also the amount of food they could afford.

*“The second time I went to the shop… I bought less, so instead of 2 packages of milk, just one… same for yoghurt… And still, I needed to pay a lot of money… So, if I go to the shop now… I don’t buy milk, juice or yoghurt; I buy only one kind of fruit like bananas… I try to go not that much to the shop”* (Man, 21 years, from South America).

European students appeared to be less financially restricted than some of the non-European international students. For European students, the increased cost did not appear to necessitate giving up staple foods, such as bread and milk; however, they also seemed to have a less varied diet.

*“Of course, it would be nice if food were cheaper, but anyway... (…) Here I eat at the restaurant of the school, but not too much… It’s cheaper to make my own meals… (…) …I also always try to buy the cheapest products...”* (woman, 20 years from Europe).

Among some European students, high prices appeared to lead to positive changes in the health impacts of their food choices.

*“Back home I would eat a lot of chips and chocolate, but once again it’s really expensive, two or three times the price I would normally pay so it’s not worth it to buy it.”* (man, 19 years from Europe).

This was also the case for alcohol, which is very expensive in Norway, partly because of high taxes.

### 3.2.2. Coping strategies

The international students in this study described various coping strategies to deal with the high cost of food in addition to restricting their shopping. Seven out of the ten participants reported buying foods on discount or eating out only when there were special offers.

*“Here in the shops you sometimes also get a discount on products that are about to expire ... You can find them in a box in the shop. That’s a good thing! I like that! But most of the time I don’t know the products.”* (man, 29 years from Europe).

Half of the participants, including four Europeans, stated that they became more cautious about food waste because of price concerns.

*“I’m not throwing away food here ... I eat everything I can... At home I’m not cooking and buying food so ... there I throw away more food because I don’t realize that it costs money ... I don’t feel the pain, you know ... Here I need to pay for my own food ... So, I keep it or eat it!”* (woman, 20 years from Europe).

Additionally, one European participant even started “dumpster diving” to retrieve food discarded by grocery stores.

*“I once went to look with my German roommate in the stuff that the shops throw away ... And there was a plastic box with grapes, we opened it, and only half of the grapes was bad. So, the other grapes we took home ... We also found some brussels sprouts. Another time I went there, and I found a plastic box with trashed carrots”* (man, 29 years from Europe).

The only non-European student who mentioned food waste made himself eat food he did not like to reduce waste.

*“I will not buy that product again...(…) you still must eat it. I try to not just throw it away; I will share the food with friend who likes it..., or…at least I will finish it...”* (man, 21 years from Asia).

Traveling by ferry to Denmark for cross-border shopping is a popular activity for locals in Southern Norway due to significant price differences in alcohol, tobacco, and meat in particular. Four of the participants in this study, three of whom were European, adopted this custom.

*“I sometimes take the boat to Denmark to buy alcohol. There is a limit on the amount you can bring, I think eighteen or nineteen beers, so I just brought that back home.”* (man, 19 years from Europe).

For the majority of the participants, increased food costs led to a less varied diet. Many resorted to buying less food or opting for cheaper options that they would not usually choose, and some devised more creative ways to obtain inexpensive or free food.

## 3.3. Theme two: Missing the taste from home

Most participants expressed a dislike for foods in their host country and perceived food from home to taste better and be of higher quality. Several international students explicitly stated that they missed food from home, and all participants reported that they tried to find food that was familiar from their home countries.

### 3.3.1. Food from home is better

There were no clear differences between Europeans and non-Europeans regarding their preferences for food from their home country and their perception of it as superior to foods in their host country.

*“The horse sausage is the best where I live. I tried sausages here, but they are not as good as in my country!”* (man, 21 years from Asia).

Participants particularly commented on the flavour of foods being less appealing and satisfying than similar items that they would eat at home. These preferences for foods from home seemed to lead to dietary changes for some students, while others ate local foods despite considering them inferior.

### 3.3.2. Missing foods from home

Several participants attempted to replicate their mothers' cooking. One student reported attempting to prepare meals as her family did at home but found it difficult, leading to significant changes in her breakfast habits on weekdays.

*“At home, my mom makes this every day! … So, during the weekend when I have time, I make these dishes… Well, only the “Upma”, the other ones are too hard to make here… During the week, when I don’t have that much time, I eat some Norwegian bread with chocolate spread or jam…”* (woman, 26 years from Asia).

The perception that food from home was the best was shared by all participants in the study, and was evident across all themes.

*“…I wish I could have some rice noodles from home! You find them here, but the taste is different. So, I really miss the food from home. And I still stay here for a few months…”* (woman, 24 years from Asia).

While some developed strategies to create a sense of home and comfort by cooking and sharing foods from home, others did not employ such strategies.

*“I live with another girl from my country, and so we understand each other perfectly! We try to cook what we eat like at home ...”* (woman, 20 years from Europe).

## 3.4. Theme three: Food literacy

In the current study, the majority of participants, both Europeans and non-Europeans, struggled to navigate the food environment in their new country because of a lack of knowledge and skills.

### 3.4.1. Lack of knowledge

While most students reported primarily eating dishes familiar from their home country, both European and non-European students expressed willingness to try new foods. However, they lacked knowledge on how to use Norwegian food products.

*“A few days ago, I was following this Norwegian guy in the supermarket, because I was interested in what he was buying ... And he bought some really weird food packed in tubes, and I didn’t know what it was ... So, I bought it, and I still don’t know what it is, but I like it and ... Well, if I could taste some typical Norwegian food, I would really like that ...”.* (man, 24 years from Europe).

Although this lack of knowledge prevented some students from purchasing, cooking, and consuming these foods, others, struggling with the unfamiliarity of Norwegian food products, sought advice and help from local students to make these purchases.

*“I didn’t dare to buy milk, ha-ha. After two weeks, I went to the shop with a Norwegian friend, and she helped me with picking the milk with the most fats … It’s not as fresh as home, but it’s okay!”.*

(woman, 26 years from Asia).

Most of the participants expressed a desire to learn more about Norwegian food culture and acknowledged their limited knowledge. One student expressed a wish to bring back some of the culinary specialties of the host culture after the exchange, but had not yet succeeded.

*“Actually, I want to learn more about the Norwegian food culture… I don’t know anything about it… When I go back home, I wish I could make some Norwegian food to serve this to my family to show it to them…”*

(man, 21 years South America).

Overall, international students struggled to identify, prepare, and use many local products available in supermarkets. Lack of knowledge about available foods and Norwegian food culture was a challenge for most students, although many were eager to learn.

### 3.4.2. Lack of skills

Lack of skills was another food-related challenge faced by some participants, as they described having little or no cooking experience before arriving in Norway.

*“At home, I live with my grandmother and she is cooking for me, so I haven’t cooked so much before I came here. ... so, I’m trying to cook nice meals.”* (man, 29 years from Europe).

In addition to these cooking challenges, one student reported experiencing difficulties with utensils upon arrival.

*“In my home country we use chopsticks… For me, it’s very difficult to eat with a fork. In the canteen here I need to eat with a fork…”* (woman, 24 years from Asia).

Nevertheless, several students expressed a desire to improve their cooking skills after having observed locals preparing unfamiliar dishes.

*“I have some roommates and one of them is Norwegian; it’s nice to see what he cooks… If I knew what to cook with strange food… I would really like that!”* (man, 24 years from Europe).

Some students took this a step further by acquiring new skills from their peers.

*“I really like cooking! At home I sometimes made eggs, so before I came here, I couldn’t really cook ... But with my roommate is going great! I’m learning a lot…”* (woman, 20 years from Europe).

Participants reported a general lack of cooking skills, especially when preparing unfamiliar foods from their host country. For some, learning from others was a strategy to make the most of their situation. Participants expressed an interest in learning both how to make traditional dishes and how to prepare cheap, healthy meals with available ingredients.

## 3.5. Theme four: Language barriers

All the participants described experiencing difficulties with the language of their new country. As described in other aspects of Theme 3, participants found it challenging to identify food products in the shops. Language barriers compounded these difficulties and restricted their food choices. To address this, they adopted various strategies to navigate their new food environments.

### 3.5.1. Using visual cues

Most students described choosing products based on their visual familiarity with the food items. A few students indicated that they examined food packaging to decide whether to purchase a product.

*“Well, because of the different languages, in the beginning, I couldn’t really read what was on the box. So, I just looked for pictures on the box.”* (woman, 21 years from Europe).

However, this strategy does not always yield desired results. For some students, relying on familiar foods from their home environment and making visual comparisons led to the purchase of incorrect products.

*“Once I bought a product in a box that looked like bacon, so I thought it was bacon ... I bought it, and when I opened it at home, it was more like “liver paste” with some bacon. So, if I knew it was liver paste, I would not buy it.”* (man, 29 years from Europe).

### 3.5.2. Using a translation tool

Most students used translation tools such as smartphone applications or Google Translate to overcome language differences.

*“So, the first weeks I was a bit lost ... And with the different language it’s even harder to find the food you look for... sometimes we use Google translate ... You can film the product with a camera, and it translates really quick! I didn’t know that the first two weeks.”* (woman, 20 years from Europe).

These tools helped students identify foods and broaden their choices beyond familiar items or guessing based on appearance. However, not all students used these tools. Some found them burdensome. Figuring out food items and nutrient content, even with a translation tool, was too time-consuming. This often led them to give up on their efforts and purchase familiar and convenient options.

*“When I’m home normally, I check the nutrients, or buy brands with less sugar and fat ... That’s something I don’t do here ... It would take too much time, and as I said, I don’t like to translate.”*

(man, 24 years from Europe).

One student reported that bringing food from her home country, not a translation tool, helped to reduce the challenges of food shopping.

*“So, when I came here… I brought a suitcase of 23 kg full of food with me… Well, let me say that I was happy that I brought all the basic food that I need with me! The language is a real difficulty for me to buy products”* (woman, 26 years from Asia).

Overall, language barriers were a significant initial challenge, but most students quickly resolved these issues by using Google Translate or similar apps. However, some students continued to struggle without embracing these tools.

## 3.6. Theme five: Social aspects of eating

Seven out of ten students said that they missed the social aspects of eating during their student exchanges. Their experiences varied depending on the students' country of origin. Non-European students, especially those from cultures where meals are communal, reported struggling more than their European counterparts did.

### 3.6.1. Missing social eating

Several European students reported that their limited budget restricted their ability to participate in social eating.

*“That’s what I miss from home ... I usually go out a lot to have a lunch or a dinner ... And here I’m like: No, no, it’s expensive ... You’re paying on your own for everything so control yourself ...”*

(woman, 20 years from Europe).

For non-European students, mealtimes were perceived as family time, and they reported feeling isolated while eating alone in their new environment.

*“It’s also strange here in Europe, that everyone cooks for themselves… Normally eating is a social event, with a lot of people together… Here I make my own dinner, and I eat alone… I only drink tea with my roommates sometimes…”* (man, 21 years from Asia).

Their food behaviours at home were closely linked to family, togetherness, and caring, making solitary eating in their new environment particularly difficult.

*“For lunch, my mother stands in the kitchen all morning to prepare the lunch… When it’s ready, every one of the family sits together on the table, and we eat and talk… Here for lunch… I eat some spicy pasta, most of the time very quick because the next class is starting already… You really can’t compare it…”* (woman, 26 years from Asia).

### 3.6.2. Social eating events

International students reported appreciation for the few social eating events organized by the university. For example, weekly free waffle hours were highly valued as both an introduction to Norwegian food culture and an opportunity to socialize.

*“The free waffle hour at the university! I also eat waffles there! I’m not doing that at home ... the free waffle hour is nice to socialise... And you get food for free... That is even more nice! Free food in Norway... So, we go and take a lot of waffles...”* (woman, 20 years from Europe).

Overall, eating alone was common among international students, and was generally perceived as negative. Non-European students, in particular, reported missing the social aspects of sharing meals with friends or family and appreciated food-related social events organized by the university.

# 4. Discussion

Limited data are available on dietary acculturation among international students from outside English-speaking Western countries, including those in the Nordic region, and how it affects their health and well-being. This qualitative study aimed to gain insight into the main challenges international students face concerning food choices and eating habits after encountering Norwegian food culture. International students struggled in various ways to adapt to Norwegian food culture, which, for some, had a negative impact not only on their diet but also on their social and emotional well-being. Food costs appear to be a concern for everyone and may lead to unfavourable consequences, especially for the most vulnerable, less financially robust students from non-European countries. Several, but not all, participants appeared lonely and missed eating together with others. Low food literacy competencies and language barriers contributed to altered dietary habits, and sometimes to the detriment of students’ diet quality. The most common negative changes in food habits observed in this study were eating a less varied diet or eating alone. Several out of these negative impacts observed in this study seemed to affect the students’ well-being.

## 4.1. Food costs – food security

Food costs affected all participants, but the impact of high prices varied significantly among the students. For some, it led to improvements in diet quality, as they omitted unhealthy and nutritionally unnecessary foods, such as chocolate. In contrast, some non-European students responded to high food costs by ceasing to purchase nutritious staples, such as milk. This behaviour led to restricted dietary intake and a lack of variety, indicating that some international students may be experiencing food insecurity. Food insecurity is defined as the inability to secure regular access to enough nutritious and safe food or obtain adequate food in a socially appropriate manner (Bickel et al., 2000). This indication that some students in this study were food insecure may be explained by the fact that, according to 2023 data, the costs of food and non-alcoholic beverages in Norway are among the top three highest in Europe—along with Iceland and Switzerland—at 130% compared to the EU average (Eurostat, 2024).

In recent years, food insecurity in the general student population has gained increasing attention. A 2017 review highlighted that food insecurity is surprisingly prevalent among students, with mean rates of 35–42% in the US and other countries, including Canada, South Africa, New Zealand, Australia, Malaysia, and Mexico (Bruening et al., 2017). Recent data from Australia and the US indicate that international students are more prone to experiencing food insecurity than their domestic peers (Dana et al., 2023; Glick et al., 2025). Food insecurity is associated with poor health outcomes (Bruening et al., 2017) and adverse academic outcomes (Hagedorn et al., 2019), highlighting the need to address this issue.

Data on food insecurity among students in general, including international students, in Nordic countries are limited. However, a case report from Norway documented severe weight loss among an already-lean international student from a non-European country due to limited food intake (Halvorsen et al., 2005). Moreover, a Norwegian qualitative study investigated aspects of food security among international students, showing that high food prices and limited variety in grocery stores hinder their ability to meet dietary preferences and maintain a diverse diet (Bauch et al., 2023). Conducting a survey among international students in Norway with questions on food insecurity would be valuable for further exploring the prevalence of this issue.

## 4.2. Missing foods from home

In this study, students, regardless of their nationality, perceived food from their home countries as superior in terms of taste, texture, and quality, compared to foods in Norway. This observation aligns with research on international students in the UK and Canada, which showed that they held negative perceptions of food in their host country and preferred traditional foods from their home cultures (Amos & Lordly, 2014; Brown et al., 2010; Brown et al., 2019). The preference for home foods and dietary patterns is associated with the need for comfort, and cultural and religious customs (Brown et al., 2019). The participants in our study described home-food in relation to comfort and feelings of family connectedness. For some, preferences for foods from home may reflect missing home rather than not liking the food in their new country, and cooking dishes from home seems to provide students with something familiar, comforting, and connectedness. This finding aligns with a US study, which indicated that while international students miss their families and cultural food environment back home, maintaining their traditional eating habits and culinary practices is essential for preserving their bond to their home culture and family, as well as for their overall well-being (Wright et al., 2021).

Although several participants in our study showed attachment to home foods and experienced some degree of neophobia, they expressed dissatisfaction with not knowing how to use Norwegian ingredients, and many demonstrated an interest in and openness to new foods. This corroborates findings from the UK, which indicate that international students are open to tasting and trying new foods despite their preference for foods from their home countries (Brown et al., 2010). To accommodate these preferences and the need for familiarity and support observed among some international students, universities could offer simple cooking courses combined with shared meals. This could introduce students to the local food culture and methods for cooking both new and familiar, affordable, and nutritious foods, while simultaneously improving the well-being of all international students, including those most vulnerable. Such approach is supported by data indicating that social eating acts as a mechanism for facilitating social bonding, rather than resulting from it (Dunbar, 2017).

## 4.3. Food literacy

Food literacy, defined as ‘the tools needed for a healthy lifelong relationship with food’ (Vidgen & Gallegos, 2014, p. 54), encompasses both the knowledge and skills needed to navigate the food system and meet dietary recommendations. In the current study, several international students encountered challenges in adjusting to a new food culture and environment, indicating that they were not sufficiently food-literate. While some of them did not encounter significant difficulties, it was evident that both non-European and European students wanted more guidance, including in cooking classes. For some, this was out of curiosity, while for others, it seemed to be out of necessity.

Being insufficiently food literate and experiencing challenges related to budgeting, planning, and cooking nutritious dishes that are both affordable and acceptable is not unique to international students (Slater et al., 2018). Studies among the broader student population have demonstrated struggles during this important transition period in their lives, with evidence suggesting that those who are more food literate cope better (Blichfeldt & Gram, 2013).

However, the findings among the international students in this study indicate that the relationship between food literacy and diet quality may also be linked to issues of food insecurity. A US study involving college students supports these findings, showing the positive effect of a program aimed at increasing food literacy, with indications that this may lead to a reduction in food insecurity (Morgan et al., 2023). Additionally, several international studies have consistently shown a positive correlation between various aspects of food literacy and diet quality (Akkartal & Gezer, 2020; Brunner et al., 2010; Groufh-Jacobsen et al., 2023; Murakami et al., 2024). Collectively, these findings suggest that enhancing students’ food literacy is likely to improve their diet quality, with potentially similar effects for international students. This is furthermore supported by a study from Canada demonstrated that biweekly community cooking and nutrition education workshops improved international students’ food literacy and sense of community (Luongo et al., 2018). Despite the limitation of the current study’s generalizability, we argue that offering cooking courses may be a promising strategy to increase food literacy while enhancing the well-being of students in general, as well as international students, particularly if combined with social events centred around food, as longed for by some participants in this study.

## 4.4. Language barriers

Language was a challenge for most students in the current study. Challenges related to language, in general, have been observed by others. A study from the US reported that international students face language barriers that hinder efficient communication with peers and university staff (Wu et al., 2015). Upon arrival in Norway, the language barriers challenged international students in the current study when adapting to their new food environment. However, over time, most students developed strategies such as using Google Translate and other digital aids to overcome these barriers. European students, in particular, seemed to navigate well from the beginning because of their English proficiency. Although several international students in the current study would probably benefit from additional language support, language challenges did not appear to be as critical as some of the other food-related challenges that students in our Norwegian context did not overcome as easily.

## 4.5. Social aspects of eating

Cooking and eating alone were common among the participants in this study. Solitary eating seemed to be especially difficult to adapt to for some of the non-European students, coming from cultures where food is shared, and eating is fundamentally a social event. This finding is not surprising, given the substantial body of evidence demonstrating that people generally find social eating more enjoyable than eating alone, and that it has a positive impact on their well-being (Herman et al., 2019, p. 216). A larger study conducted in the Nordic countries shows that in 2012, 41% of meals in Norway were eaten without the company of others. Eating alone was more common in one-person households. Additionally, significant variation was evident across different meal types: dinner and lunch were the meals least likely to be eaten alone, whereas more than half of breakfasts and in-between meals were typically consumed alone (Holm, 2016). Studies on solitary eating across different cultures reveal significant variations. Early 2000s data indicate a tendency in the US towards more solitary eating, comparable to the Nordic data, whereas a significantly lower prevalence of solitary eating has been observed in the Netherlands (Pliner & Bell, 2009). Similarly, in non-Western contexts, significant cross-cultural variations are evident. For instance, among university students, solitary eating prompted more negative emotions for Koreans compared to Japanese students, highlighting cultural differences in the social significance of meals (Cho et al., 2015). For international students from cultures where eating alone may not be considered a proper meal, adjusting to the more individualized eating habits typical in the Nordic countries can be challenging.

Studies have found that solitary eating is associated with suboptimal nutrition in both younger and older individuals (Pliner & Bell, 2009). Eating alone has also been shown to affect food intake to a greater degree than the physiological cues of satiety and hunger (Herman et al., 2003). Furthermore, an Asian cohort study with nearly 40,000 participants observed that the act of eating together, or commensality, is important not only for diet quality and positive health outcomes, but also for social interaction and increased happiness (Yiengprugsawan et al., 2015). This seems to be reflected in how much the international students in the current study appreciated the few social eating events organized at the university. These findings, despite the limitation of the generalizability of the current study sample, suggest that promoting commensality and ideally incorporating joint cooking activities to enhance cooking skills and food literacy should be prioritized in support initiatives for international students in particular, though they would likely also benefit the general student population.

## 4.6. Strengths and limitations

This study presents a unique exploration of international students’ encounters with and adaptations to Norwegian food culture. While the interviews were limited to 20-30 minutes per participant, and the interpretation of the data does not encompass all aspects of dietary acculturation or the potential challenges associated with it — and represents just one of many possible perspectives — several measures were undertaken to ensure a transparent and rigorous approach to the data collection and analysis. These measures included using a structured interview guide focusing on key themes, encouraging participants to share detailed narratives, and employing three researchers to analyse and iteratively discuss the data, codes and themes. Additionally, the paper was co-authored by researchers from multiple countries to enhance the diversity of the perspectives represented.

It is important to acknowledge that the outcome of any interview-based qualitative study is influenced by the interviewer's perspective (Hewitt, 2007). In this study, all interviews were conducted by M.E.M.P., who, as an international student, had an in-depth understanding of the environment in which the participants lived. This facilitated participants to open up quickly, as they perceived her as a peer, which supported a relaxed interview atmosphere and ultimately resulted in rich data. However, as an international student from Europe, she may have been particularly attuned to the experiences of fellow European students. This shared cultural background likely enhanced her understanding and responsiveness to European participants' narratives, while cultural distance with non-European participants may have influenced question framing, sensitivity to cues, and interpretation of responses, potentially leading to deeper engagement with European perspectives. All interviews were conducted in English, which may have posed a barrier for some participants in articulating the full nuances of their lived experiences. However, all participants met the minimum B2-level (upper-intermediate) English proficiency requirement set by the host institution. Although M.E.M.P. had no prior experience conducting qualitative interviews, she received training in communication skills and was supported by an experienced research team.

We also acknowledge that prior research highlighting dietary challenges among international students may have influenced both the design of the interview guide and our analytic lens. While some positive experiences were present in the data, the overall emphasis on challenges likely reflects both participants’ narratives and this pre-existing focus, which may have affected our development of themes.

The aim of this study was not to achieve generalizability or to conduct an exhaustive comparison of international students from different cultures, but rather to capture a diverse range of experiences among international students in Norway. However, recruiting through convenience sampling at social events presents a limitation, as it may have favoured participation from students who are already more socially integrated, potentially excluding perspectives from more isolated students whose experiences might differ.

The issues identified during the interviews highlighted several areas that could be explored further. Specifically, it would be valuable to conduct a larger quantitative survey among international students in Norway to examine the prevalence of food-related challenges, including food insecurity, and to identify characteristics of the most vulnerable students whom future interventions should target. Future research should also investigate whether similar challenges occur in other European countries, outside the UK.

# 5. Conclusions

Findings from this qualitative study indicate that international students in Norway face several food-related challenges, which may negatively impact not only their diet, but also their social and emotional well-being. This study provides novel insights, suggesting that international students in a Nordic context could benefit from interventions targeting food literacy and their social food environment. Such interventions could help them to better manage high food costs while maintaining a diet that is both acceptable and of sufficiently high quality, while also strengthening social aspects of eating, which seems important for their well-being. Further research is needed to better understand the prevalence of the challenges identified in this study and to develop interventions that can support the most vulnerable international students, in Norway and in other countries.

# Ethics Declarations

## Ethics approval and consent to participate

The study was conducted in accordance with the Declaration of Helsinki and approved by The Norwegian Centre for Research Data (reference number: 923 564) and the Ethical Board the Faculty of Health and Sport Sciences at the University of Agder, Norway (reference number: RITM0064045). Informed consent was obtained from all subjects involved in the study.

## Generative Artificial Intelligence (AI)

The authors have written the entire text. This was then proofread and improved for clarity using UiO GPT (OpenAI's GPT-4 Omni) through the University of Oslo, accessed via the University of Agder in February, June, October and November 2025. The tool did not alter the content, and ideas, information, and the meaning remained unchanged. No modifications were made to these tools. The authors declare that they have no competing interests related to its use.

## Consent for publication

Not applicable.

## Competing interests

No potential competing interest was reported by the authors.

## Funding

This research received no external funding.

## Authors' contributions

Conceptualization, A.C.M. and N.C.Ø.; investigation, M.E.M.P.; formal analysis, K.V., A.C.M., M.E.M.P., N.C.Ø., M.E.B. and S.T.S.; writing—original draft preparation, A.C.M., N.C.Ø. and M.E.M.P.; writing—review and editing, N.C.Ø. K.V., M.E.M.P., M.E.B. and S.T.S.; project administration, A.C.M. All authors are accountable for all aspects of the work and have read and approved the final manuscript.

## Acknowledgements

Not applicable.

## Data availability statement

The data that support the findings of this study are available from the corresponding author, A.C.M., upon reasonable request.

# References

Akkartal, Ş., & Gezer, C. (2020). Is Nutrition Knowledge Related to Diet Quality and Obesity? *Ecol Food Nutr*, *59*(2), 119-129. <https://doi.org/10.1080/03670244.2019.1675654>

Alakaam, A. A., Castellanos, D. C., Bodzio, J., & Harrison, L. (2015). The factors that influence dietary habits among international students in the United States. *Journal of International Students*, *5*(2), 104-120. <https://doi.org/10.32674/jis.v5i2.428>

Amos, S., & Lordly, D. (2014). Picture this: A photovoice study of international students’ food experience in Canada. *Canadian Journal of Dietetic Practice and Research*, *75*(2), 59-63. <https://doi.org/10.3148/75.2.2014.59>

Arthur, N. (2017). Supporting international students through strengthening their social resources. *Studies in Higher Education*. <https://doi.org/10.1080/03075079.2017.1293876>

Bauch, C., Torheim, L. E., Almendingen, K., Molin, M., & Terragni, L. (2023). Food Habits and Forms of Food Insecurity among International University Students in Oslo: A Qualitative Study. *International journal of environmental research and public health*, *20*(3), 2694. <https://doi.org/10.3390/ijerph20032694>

Bickel, G., Mark Nord, Cristofer Price, William Hamilton, and John Cook (2000). *Guide to Measuring Household Food Security, Revised 2000.* <https://doi.org/10.22004/ag.econ.337157>

Blichfeldt, B. S., & Gram, M. (2013). Lost in Transition? Student food consumption. *Higher Education*, *65*(3), 277–289. <http://www.jstor.org/stable/23351697>

Braun, V., & Clarke, V. (2022). Conceptual and design thinking for thematic analysis. Qualitative Psychology, 9(1), 3–26. <https://doi.org/10.1037/qup0000196>

Braun, V., & Clarke, V. (2006). Using thematic analysis in psychology. Qualitative Research in Psychology, 3(2), 77–101. <https://doi.org/10.1191/1478088706qp063oa>

Brown, L., Edwards, J., & Hartwell, H. (2010). A taste of the unfamiliar. Understanding the meanings attached to food by international postgraduate students in England. *Appetite*, *54*(1), 202-207. <https://doi.org/https://doi.org/10.1016/j.appet.2009.11.001>

Brown, L., Farbrother, C., & Dazam, J. (2019). Longing for a taste of home. *British Food Journal*, *121*(12), 3281-3292. <https://doi.org/10.1108/BFJ-03-2019-0179>

Bruening, M., Argo, K., Payne-Sturges, D., & Laska, M. N. (2017). The Struggle Is Real: A Systematic Review of Food Insecurity on Postsecondary Education Campuses. *Journal of the Academy of Nutrition and Dietetics*, *117*(11), 1767-1791. <https://doi.org/10.1016/j.jand.2017.05.022>

Brunner, T. A., van der Horst, K., & Siegrist, M. (2010). Convenience food products. Drivers for consumption. *Appetite*, *55*(3), 498-506. <https://doi.org/10.1016/j.appet.2010.08.017>

Bugge, A. B., & Almås, R. (2006). Domestic dinner:Representations and practices of a proper meal among young suburban mothers. *Journal of Consumer Culture*, *6*(2), 203-228. <https://doi.org/10.1177/1469540506064744>

Cho, W., Takeda, W., Oh, Y., Aiba, N., & Lee, Y. (2015). Perceptions and practices of commensality and solo-eating among Korean and Japanese university students: A cross-cultural analysis. *Nutrition research and practice*, *9*(5), 523–529. <https://doi.org/10.4162/nrp.2015.9.5.523>

Dana, L. M., Wright, J., Ward, R., Dantas, J. A. R., Dhaliwal, S. S., Lawrence, B., O'Connor, M., Booth, S., Kerr, D. A., & Pollard, C. M. (2023). Food Insecurity, Food Assistance, and Psychological Distress among University Students: Cross-Sectional Survey Western Australia, 2020. *Nutrients*, *15*(11). <https://doi.org/10.3390/nu15112431>

Dean, J. A., Gallo, S., & Anderson, A. K. (2024). Factors associated with dietary acculturation among international students at the University of Georgia. *Journal of American College Health*, *72*(8), 2899-2905. <https://doi.org/10.1080/07448481.2022.2138716>

Dunbar R. I. M. (2017). Breaking Bread: the Functions of Social Eating. *Adaptive human behavior and physiology*, *3*(3), 198–211. <https://doi.org/10.1007/s40750-017-0061-4>

Eurostat. (2024). *Comparative price levels for food, beverages and tobacco*. Retrieved May 9, 2025 from: <https://ec.europa.eu/eurostat/statistics-explained/index.php?title=Comparative_price_levels_for_food,_beverages_and_tobacco>

Guest, G., MacQueen, K. M., & Namey, E. E. (2012). Applied thematic analysis. SAGE Publications, Inc., <https://doi.org/10.4135/9781483384436>

Gilbert, P. A., & Khokhar, S. (2008). Changing dietary habits of ethnic groups in Europe and implications for health. *Nutr Rev*, *66*(4), 203-215. <https://doi.org/10.1111/j.1753-4887.2008.00025.x>

Glick, A. A., Winham, D. M., & Shelley, M. C. (2025). Food Insecurity Predictors Differ for White, Multicultural, and International College Students in the United States. *Nutrients*, *17*(2). <https://doi.org/10.3390/nu1702023> 7

Groufh-Jacobsen, S., Larsson, C., Van Daele, W., Margerison, C., Mulkerrins, I., Aasland, L. M., & Medin, A. C. (2023). Food literacy and diet quality in young vegans, lacto-ovo vegetarians, pescatarians, flexitarians and omnivores. *Public Health Nutr*, *26*(12), 3051-3061. <https://doi.org/10.1017/s1368980023002124>

Hagedorn, R. L., McArthur, L. H., Hood, L. B., Berner, M., Anderson Steeves, E. T., Connell, C. L., Wall-Bassett, E., Spence, M., Babatunde, O. T., Kelly, E. B., Waity, J. F., Lillis, J. P., & Olfert, M. D. (2019). Expenditure, Coping, and Academic Behaviors among Food-Insecure College Students at 10 Higher Education Institutes in the Appalachian and Southeastern Regions. *Current developments in nutrition*, *3*(6), nzz058-nzz058. <https://doi.org/10.1093/cdn/nzz058>

Halvorsen, T. F., Medin, A. C., & Karstad, M. H. (2005). [Foreign students can not manage without food and drink]. *Tidsskr Nor Laegeforen*, *125*(16), 2228-2229. <https://www.ncbi.nlm.nih.gov/pubmed/16138146>

Hartwell, H. J. E., J.S.A.; Brown, L. (2011). Acculturation and food habits: lessons to be learned. *British Food Journal*, *113* 1393-1405. <https://doi.org/10.1108/00070701111180003>

He, R., Köksal, S., Cockayne, H., & Elliot, D. L. (2024). It’s more than just food: the role of food among Chinese international students’ acculturation experiences in the UK and USA. *Food, Culture & Society*, *28*(1), 306-324. <https://doi.org/10.1080/15528014.2024.2330180>

Heng, T. T. (2016). Different is not deficient: contradicting stereotypes of Chinese international students in US higher education. *Studies in Higher Education*, *43*(1), 22–36. <https://doi.org/10.1080/03075079.2016.1152466>

Herman, C. P., Roth, D. A., & Polivy, J. (2003). Effects of the presence of others on food intake: a normative interpretation. *Psychol Bull*, *129*(6), 873-886. <https://doi.org/10.1037/0033-2909.129.6.873>

Herman, C. P., Polivy, J., Pliner, P., & Vartanian, L. R. (2019). Effects of social eating. In H. L. Meiselman (Ed.), *Social influences on eating* (pp. 215–226). Springer. <https://doi.org/10.1007/978-3-030-28817-4_13>

Hewitt, J. (2007). Ethical components of researcher researched relationships in qualitative interviewing. *Qual Health Res*, *17*(8), 1149-1159. <https://doi.org/10.1177/1049732307308305>

Holm, L., Lauridsen, D., Lund, T. B., Gronow, J., Niva, M., & Mäkelä, J. (2016). Changes in the social context and conduct of eating in four Nordic countries between 1997 and 2012. *Appetite*(103), 358–368. <https://doi.org/10.1016/j.appet.2016.04.034>

Luongo, G., Tucker, R., Hutchinson, C., & Dhaliwal, R. (2018). The Outcomes of the Community Cooking Workshops for International Students at Simon Fraser University. *Journal of International Students*, *8*(4), 1549–1568. <https://doi.org/10.32674/jis.v8i4.218>

Migration Data Portal. (2024). *International students*. Retrieved 09 January, 2025 from <https://www.migrationdataportal.org/themes/international-students>

Morgan, M., Arrowood, J., Farris, A., & Griffin, J. (2023). Assessing food security through cooking and food literacy among students enrolled in a basic food science lab at Appalachian State University. *Journal of American College Health*, *71*(1), 30-35. <https://doi.org/10.1080/07448481.2021.1880414>

Murakami, K., Shinozaki, N., Okuhara, T., McCaffrey, T. A., & Livingstone, M. B. E. (2024). Self-perceived food literacy in relation to the quality of overall diet and main meals: A cross-sectional study in Japanese adults. *Appetite*, *196*, 107281. <https://doi.org/10.1016/j.appet.2024.107281>

Neri, L. de C. L., Fiorini, S., Bosio, F., Guglielmetti, M., Ferraro, O. E., Tagliabue, A., & Ferraris, C. (2025). Nutritional health assistance for adapting food culture to the Mediterranean pattern for international students: study protocol. *International Journal of Food Sciences and Nutrition, 76*(7), 725–735. https://doi.org/10.1080/09637486.2025.2561711

Norwegian Directorate for Higher Education and Skills. (2024). *Database for Statistics on Higher Education in Norway*. Retrieved January 10, 2025 from <https://dbh.hkdir.no/tall-og-statistikk/statistikk-meny/internasjonalisering/statistikk-side/17.2/param?visningId=123>

Oberg, K. (1960). Cultural Shock: Adjustment to New Cultural Environments. *Practical Anthropology*, *os-7*(4), 177-182. <https://doi.org/10.1177/009182966000700405>

Pliner, P., & Bell, R. (2009). 9 - A table for one: the pain and pleasure of eating alone. In H. L. Meiselman (Ed.), *Meals in Science and Practice* (pp. 169-189). Woodhead Publishing. <https://doi.org/https://doi.org/10.1533/9781845695712.4.169>

Popovic-Lipovac, A., & Strasser, B. (2015). A review on changes in food habits among immigrant women and implications for health. *Journal of immigrant and minority health*, *17*(2), 582-590. <https://doi.org/10.1007/s10903-013-9877-6>

Poyrazli, S., & Lopez, M. D. (2007). An exploratory study of perceived discrimination and homesickness: a comparison of international students and American students. *The Journal of psychology*, *141*(3), 263–280. https://doi.org/10.3200/JRLP.141.3.263-280

Redfield, R., Linton, R. and Herskovits, M. (1936). Memorandum for Acculturation. American Anthropologist.

Roy, A., Newman, A., Ellenberger, T., & Pyman, A. (2019). Outcomes of international student mobility programs: a systematic review and agenda for future research. *Studies in Higher Education*, *44*(9), 1630-1644. <https://doi.org/10.1080/03075079.2018.1458222>

Sanou, D., O'Reilly, E., Ngnie-Teta, I., Batal, M., Mondain, N., Andrew, C., Newbold, B. K., Bourgeault, I. L. (2014). Acculturation and nutritional health of immigrants in Canada: A scoping review. *Journal of immigrant and minority health*, *16*(1), 24-34. <https://doi.org/10.1007/s10903-013-9823-7>

Satia, J. A. (2010). Dietary acculturation and the nutrition transition: an overview. *Appl Physiol Nutr Metab*, *35*(2), 219-223. <https://doi.org/10.1139/h10-007>

Slater J, Falkenberg T, Rutherford J, Colatruglio S. Food literacy competencies: A conceptual framework for youth transitioning to adulthood. *Int J Consum Stud*. 2018; 42: 547–556. <https://doi.org/10.1111/ijcs.12471>

Statistics Norway. (2023). *Students in higher education*. Retrieved 10 January, 2025 from <https://www.ssb.no/en/statbank/table/09224/tableViewLayout1/>

University of Agder. (2023). *Facts and history*. University of Agder. Retrieved January 10, 2025 from <https://www.uia.no/english/about-uia/facts-and-history/>

Vidgen, H. A., & Gallegos, D. (2014). Defining food literacy and its components. *Appetite*, *76*, 50-59. <https://doi.org/https://doi.org/10.1016/j.appet.2014.01.010>

Wiers-Jenssen, J. (2019). Paradoxical Attraction? Why an Increasing Number of International Students Choose Norway. *Journal of Studies in International Education*, *23*(2), 281-298. <https://doi.org/10.1177/1028315318786449>

Wright, K. E., Lucero, J. E., Ferguson, J. K., Granner, M. L., Devereux, P. G., Pearson, J. L., & Crosbie, E. (2021). The influence of cultural food security on cultural identity and well-being: a qualitative comparison between second-generation American and international students in the United States. *Ecol Food Nutr*, *60*(6), 636-662. <https://doi.org/10.1080/03670244.2021.1875455>

Wu, H.-p., Garza, E., & Guzman, N. (2015). International Student’s Challenge and Adjustment to College. *Education Research International*, *2015*(1), 202753. <https://doi.org/https://doi.org/10.1155/2015/202753>

Yiengprugsawan, V., Banwell, C., Takeda, W., Dixon, J., Seubsman, S.-A., & Sleigh, A. C. (2015). Health, happiness and eating together: what can a large Thai cohort study tell us? *Global journal of health science*, *7*(4), 270-277. <https://doi.org/10.5539/gjhs.v7n4p270>

**Table 1.** Characteristics of the participants

| **No** | **Gender** ^1^ | **Age, years** | **Continent of origin** |
| --- | --- | --- | --- |
| 1 | Woman | 21 | Europe |
| 2 | Man | 24 | Europe |
| 3 | Man | 29 | Europe |
| 4 | Woman | 20 | Europe |
| 5 | Man | 19 | Europe |
| 6 | Man | 21 | Asia |
| 7 | Woman | 26 | Asia |
| 8 | Man | 21 | South America |
| 9 | Woman | 24 | Asia |
| 10 | Man | 24 | Asia |

^1^ Participants reported their gender as either man or woman.
